# Ameloblastic Fibrodentinoma: A Case with Varied Patterns of Dysplastic Dentin

**DOI:** 10.7759/cureus.1349

**Published:** 2017-06-14

**Authors:** Radhika M Bavle, Sudhakara Muniswammappa, Reshma Venugopal, Amulya S R

**Affiliations:** 1 Department of Oral and Maxillofacial Pathology, Krishnadevaraya College of Dental Sciences and Hospital

**Keywords:** dysplastic dentin, dentinoid, mandibular tumor, paediatric odontogenic tumors, induction, benign odontogenic tumors, ameloblastic fibrodentinoma

## Abstract

Ameloblastic fibrodentinoma is a benign odontogenic tumor belonging to the category of "odontogenic epithelium with odontogenic ectomesenchyme" along with recognition of induction in the form of dentin in atypical or dysplastic forms. The biological behaviour of ameloblastic fibrodentinoma is not very different from ameloblastic fibroma; hence, it is treated similarly by conservative procedures. It is important to understand the histopathogenesis of these rare tumors. Though rare, they are an independent entity awaiting recognition. Here, we report a case of amelobalstic fibrodentinoma in a 14-year-old female patient.

## Introduction

Odontogenic tumors are a diverse set of tumors. The recent World Health Organization (WHO) 2017 classification on odontogenic tumors categorizes odontogenic tumors as epithelial, mesenchymal, and mixed [1]. They can arise from a variety of odontogenic cells/tissues possibly from the odontogenic apparatus. Classically, mixed and mesenchymal odontogenic tumors show evidence of odontogenic induction. Most of the tumors with induction have their names certified in the WHO classification, which includes odontome, but some of them like ameloblastic fibrodentinoma (AFD) and ameloblastic fibroodontoma (AFO) that have shown induction of different varieties of dentin and both dentin/enamel, respectively, have not been recognized by the recent WHO 2017 classification as independent unique tumors and are included under the category of odontomas (developing odontomas) [1].

AFD is a benign odontogenic tumor that histomorphologically simulates an ameloblastic fibroma (AF) but can form dysplastic dentin. It predominantly presents as an intraosseous tumor in the gnathic skeleton, more common in the mandible than the maxilla. Though peripheral lesions are reported, they form miniscule numbers. It accounts for a small percentage of odontogenic tumors and only around 64 cases have been reported so far since its first description by Straith in 1936 [2-3]. Here, we present a case of ameloblastic fibrodentinoma in a 14-year-old girl with features of ameloblastic fibroma and induction in the form of different types of dysplastic dentin adding a 65th case report to the literature.

## Case presentation

**Figure 1:**
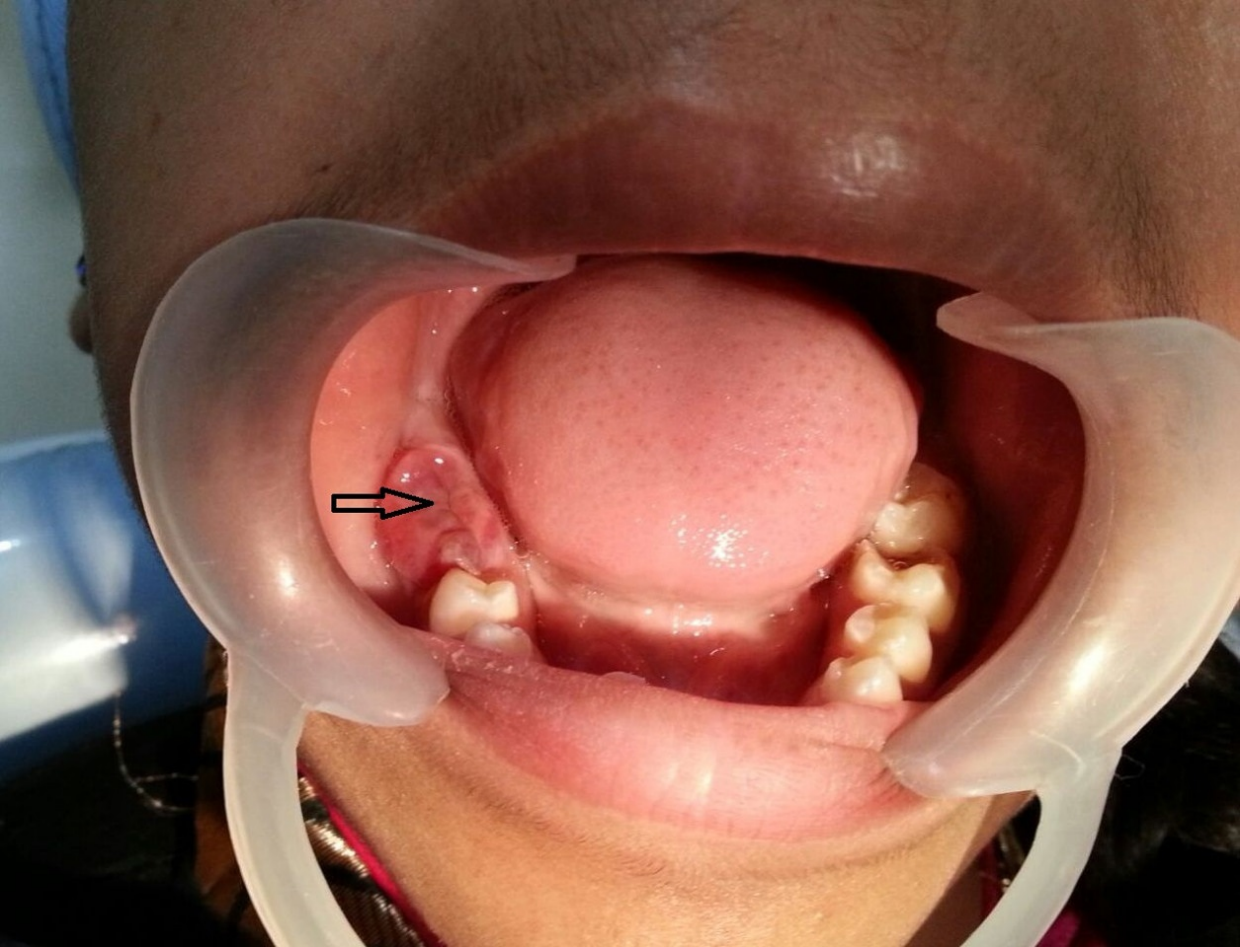
Mild erythematous swelling with the indentation marks of the opposing maxillary teeth is seen on the right posterior alveolar ridge of mandible (Arrow).

A 14-year-old female child reported to the clinical department with a complaint of non-erupted posterior teeth. There was no associated pain or paraesthesia. Medical history and family history were noncontributory. On examination, a swelling 3 x 2.5 cm involving the right mandibular alveolus was seen in the region of 43 to 48. The swelling appeared sessile, soft to firm in consistency, was slightly erythematous on the superior surface, bore the indentation marks of the opposing maxillary teeth (Figure 1) and was non-tender. The teeth 46 and 47 were missing, whereas 45 and 44 were erupted with normal morphology and were well aligned.

**Figure 2:**
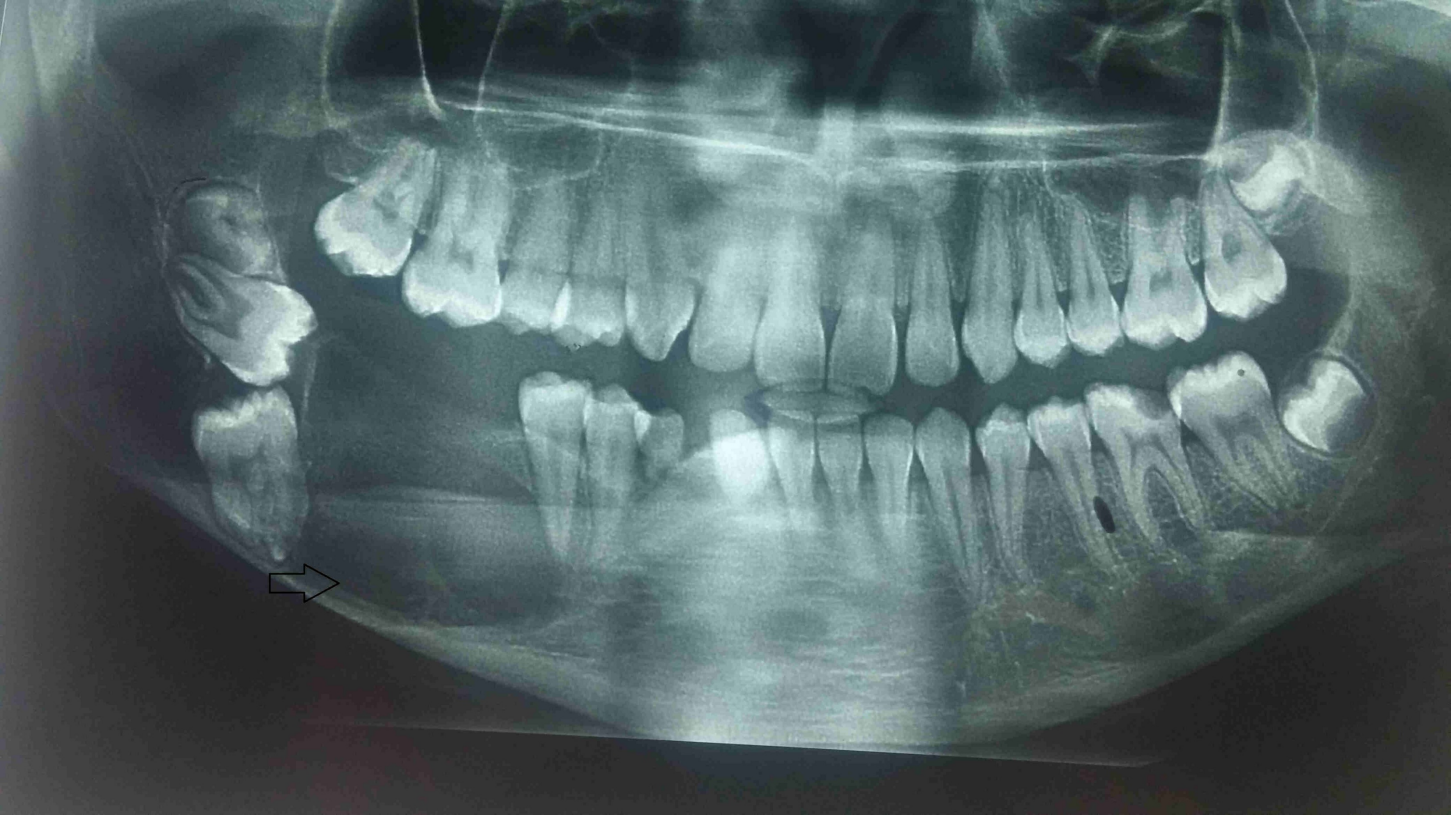
Orthopantomogram showing a well-defined unilocular radiolucent lesion extending from 44 to the retromolar area displacing and impacting 46, 47. The alveolar bone is destroyed in the 46 to 48 area, with intact lower border (Arrow).

An orthopantomogram taken revealed a well-defined unilocular radiolucent lesion in relation to 44 to 48 region. The radiolucency extended from 44 to the retromolar area displacing and impacting 46, 47 and distally displacing developing 48 into the ramus of the mandible. The mandibular right second premolar showed no resorption or displacement but was placed completely in the tumor mass. The displaced 43 was also evident. The alveolar bone was destroyed in the 46 to 48 area, but the lower border of the mandible was guarded (Figure 2). A provisional diagnosis of benign odontogenic tumor was made and an incisional biopsy was done.

**Figure 3:**
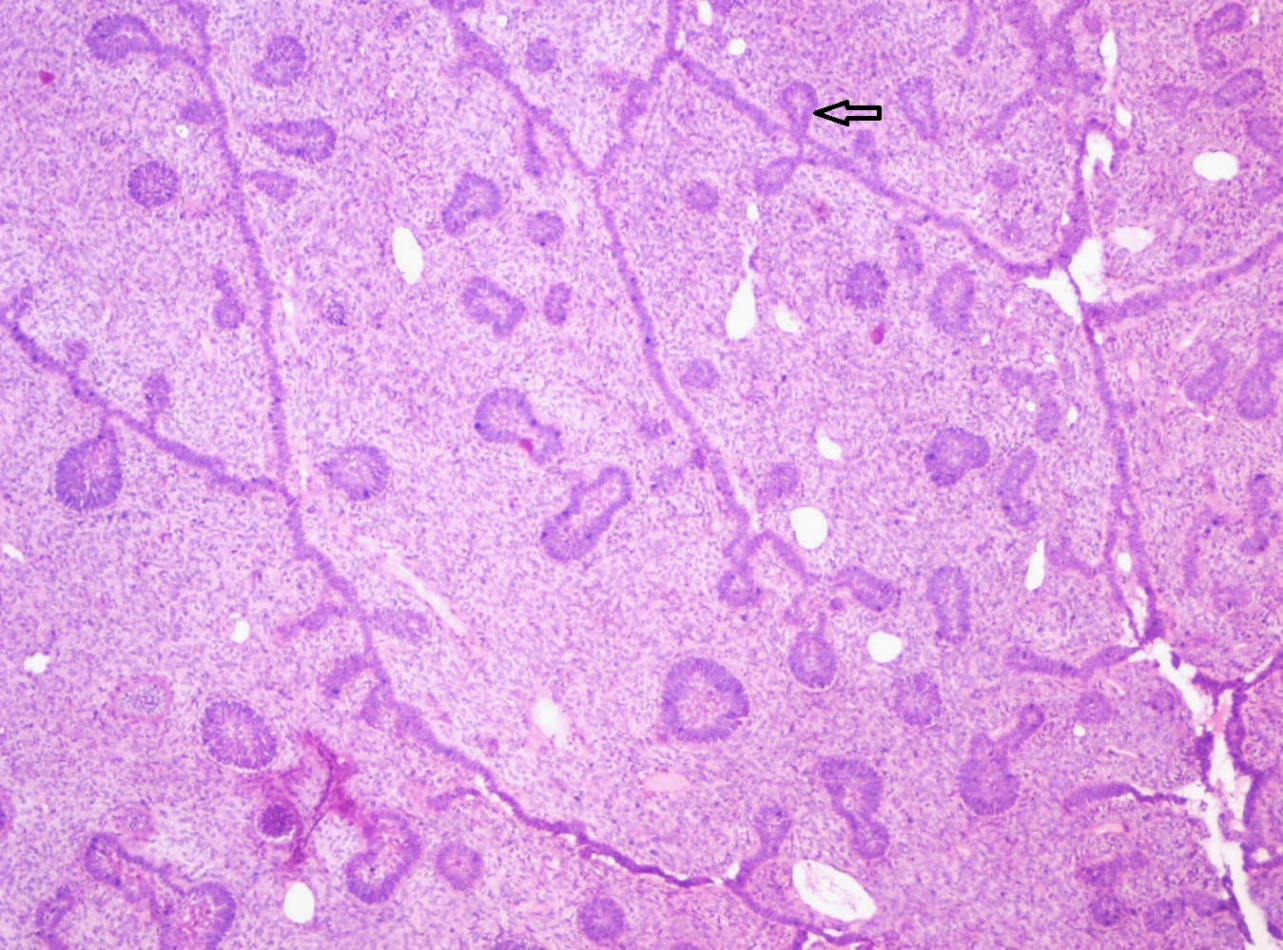
Photomicrograph showing interconnecting strands and follicles of odontogenic epithelium. Strands having knot-like branching of epithelium (Arrow) in a cell-rich primitive ectomesenchyme is evident (Haematoxylin & Eosin stain, x40).

**Figure 4:**
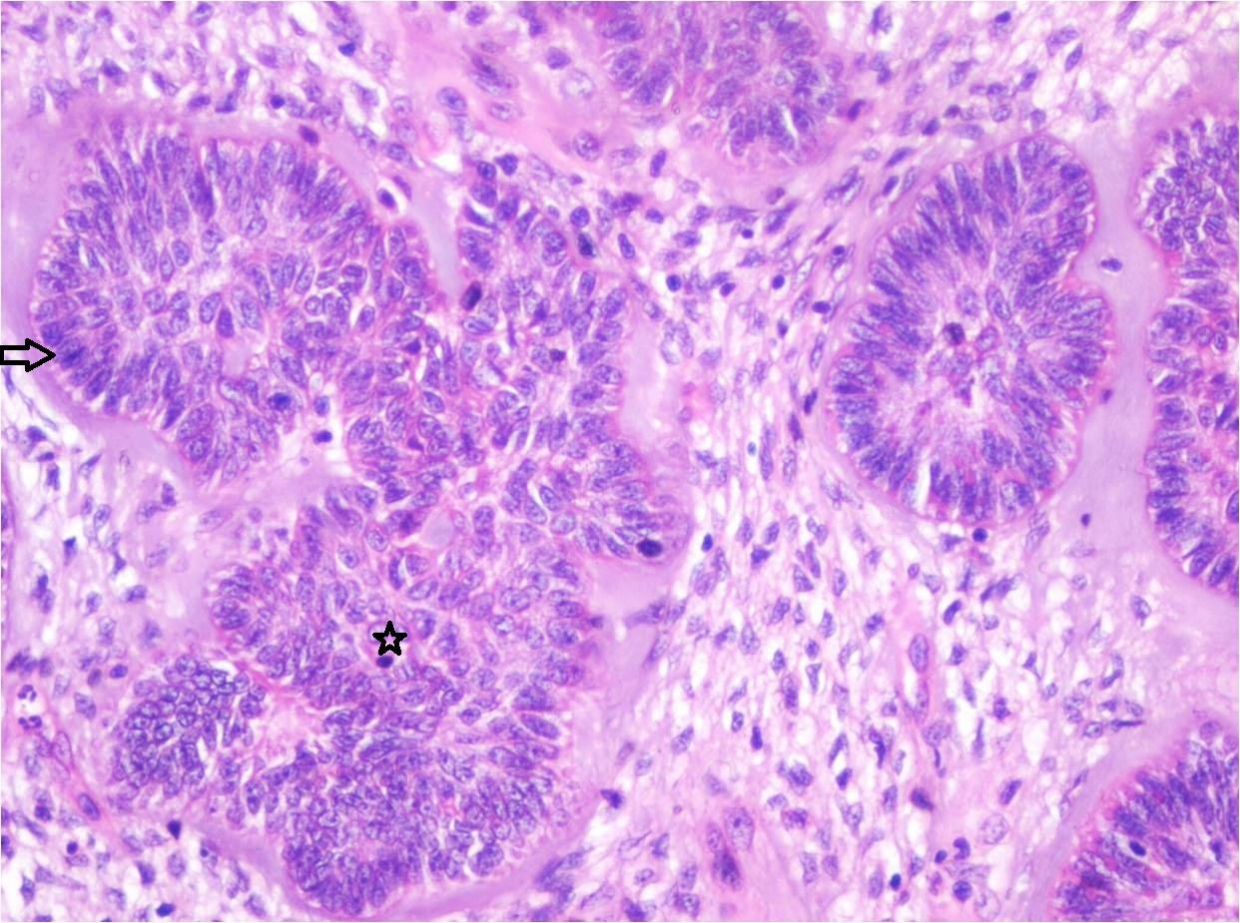
Photomicrograph showing follicles of odontogenic epithelium lined by tall columnar cells with hyperchromatic nuclei resembling ameloblasts (Arrow) and central stellate reticulum like cells (Star) in a cell-rich ectomesenchyme. Each follicle is surrounded by pale eosinophilic hyalinized area resembling dysplastic dentin-like material (Haematoxylin & Eosin stain, x200).

The biopsied tissue showed highly cellular myxoid stroma made of ectomesenchymal cells consisting of plump fibroblasts with ovoid hyperchromatic/vesicular nucleus with indistinct cell borders. Multiple branching strands of odontogenic epithelium simulating the dental lamina were noticed (Figure 3). The epithelial strands consisted of cuboidal to columnar cells with hyperchromatic nuclei. At few areas the epithelial strands were opening into small follicles with peripheral tall columnar cells and central stellate reticulum-like cells. The follicles at few areas showed basal cell hyperplasia. The small follicles of odontogenic epithelium showed the presence of a hyalinized area as a halo resembling dysplastic detinoid-like material (Figure 4). Few mitotic figures were noted. A diagnosis of ameloblastic fibrodentinoma was made.

**Figure 5:**
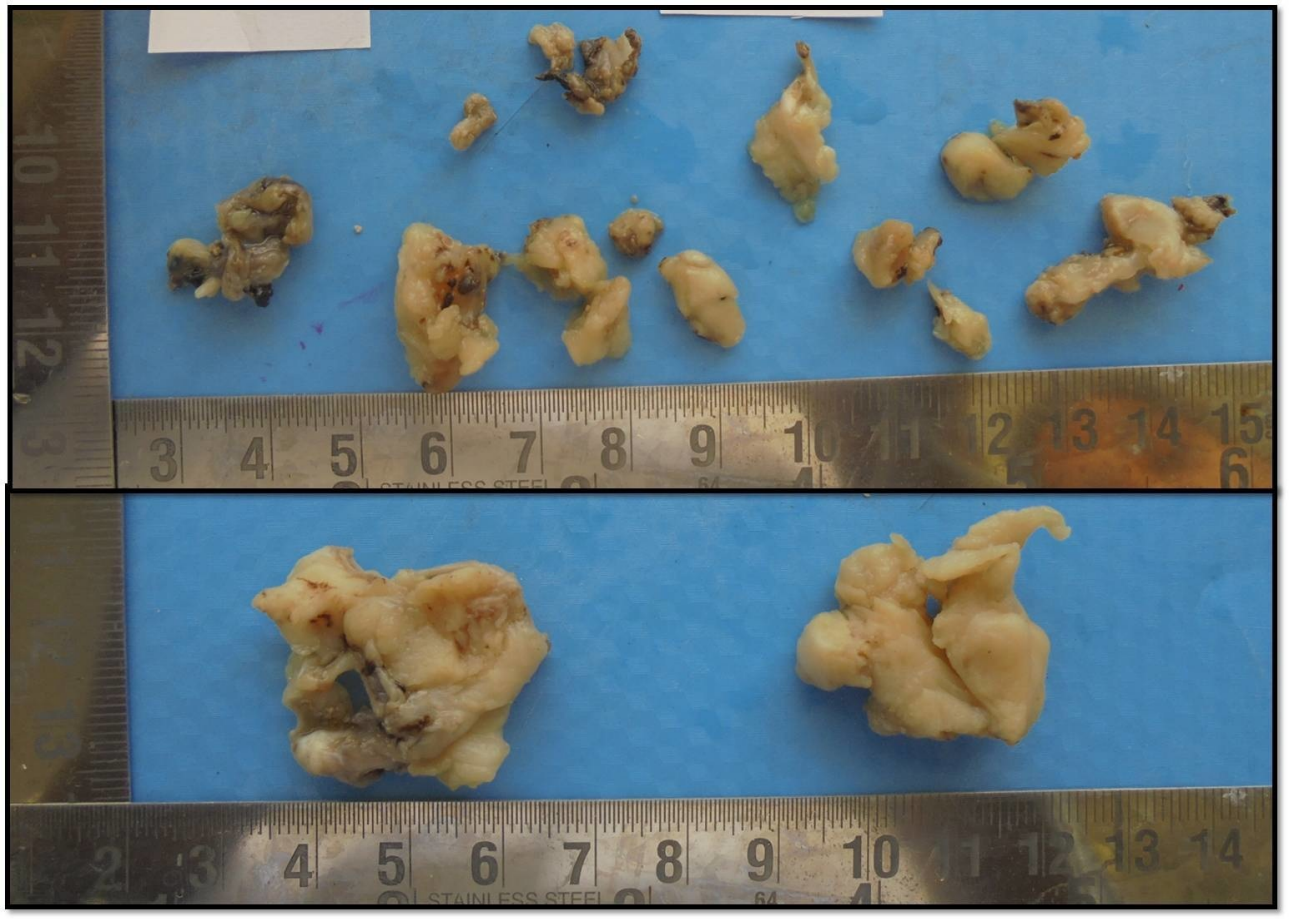
Excised tissue consisting of multiple irregular soft and hard tissue bits which appeared off-white in color.

A conservative surgical excision was carried out as the treatment for the lesion. The tumor was soft and friable; hence, it was removed in multiple bits. The tumor could be easily separated from the bony walls. The specimen was received in multiple bits and appeared off-white in color and also on cut section (Figure 5).

**Figure 6:**
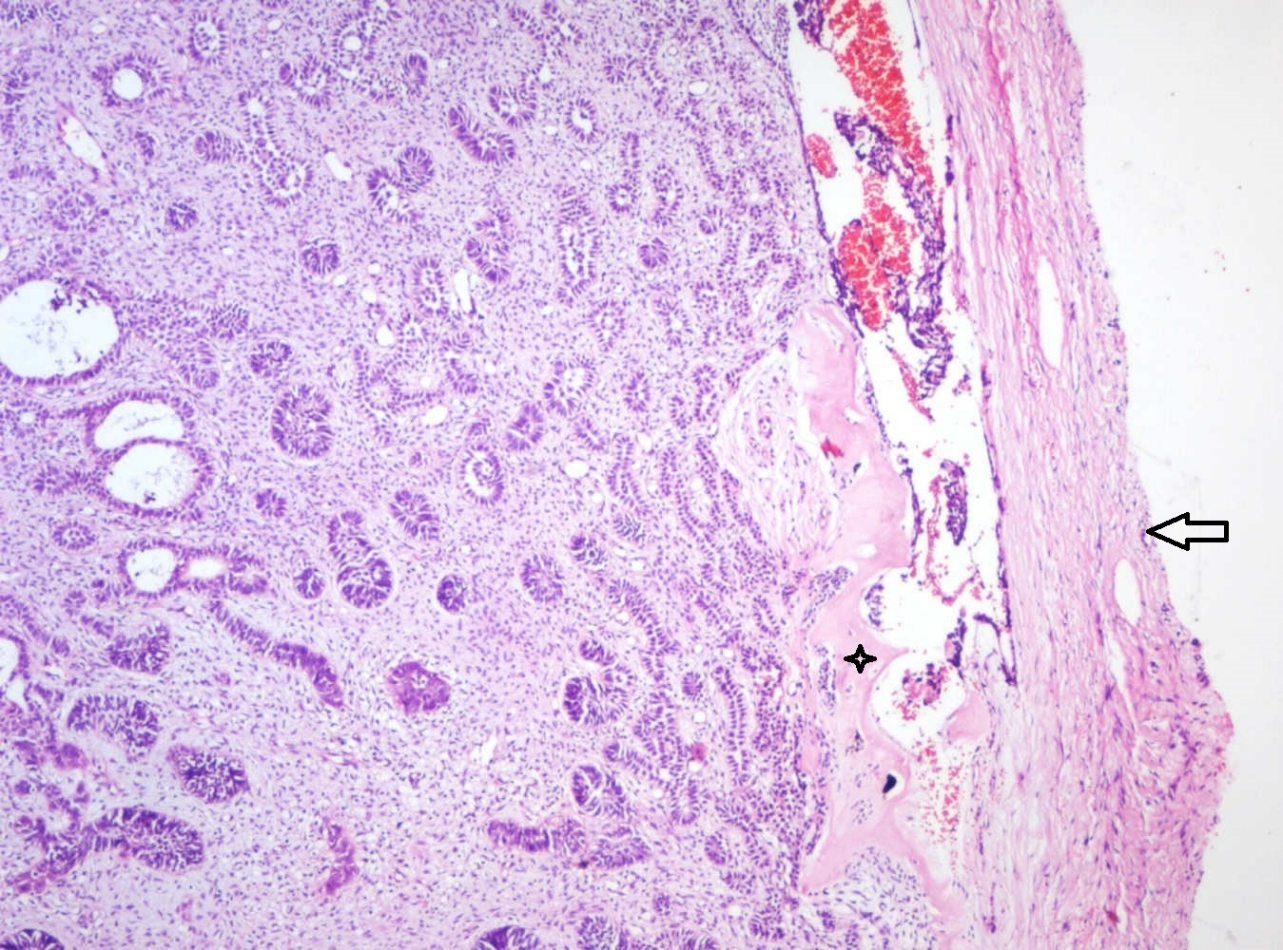
Photomicrograph of excised specimen showing multiple strands and follicles of odontogenic epithelium in a cell-rich ectomesenchyme surrounded by a fibrous capsule (Arrow). Close to the capsule, eosinophilic areas resembling dysplastic dentin (Star) is evident (Haematoxylin & Eosin stain, x40).

Histopathology of the excisional mass was similar to that of the incisional biopsy. The tumor showed the presence of a capsular structure that was incomplete. Multiple layers of collagen were seen as a capsule along with neurovascular bundles at certain areas. The tumor revealed odontogenic strands in the primitive myxoid ectomesenchymal stroma resembling an ameloblastic fibroma. Dentinoid-like material was evident (Figure 6).

**Figure 7:**
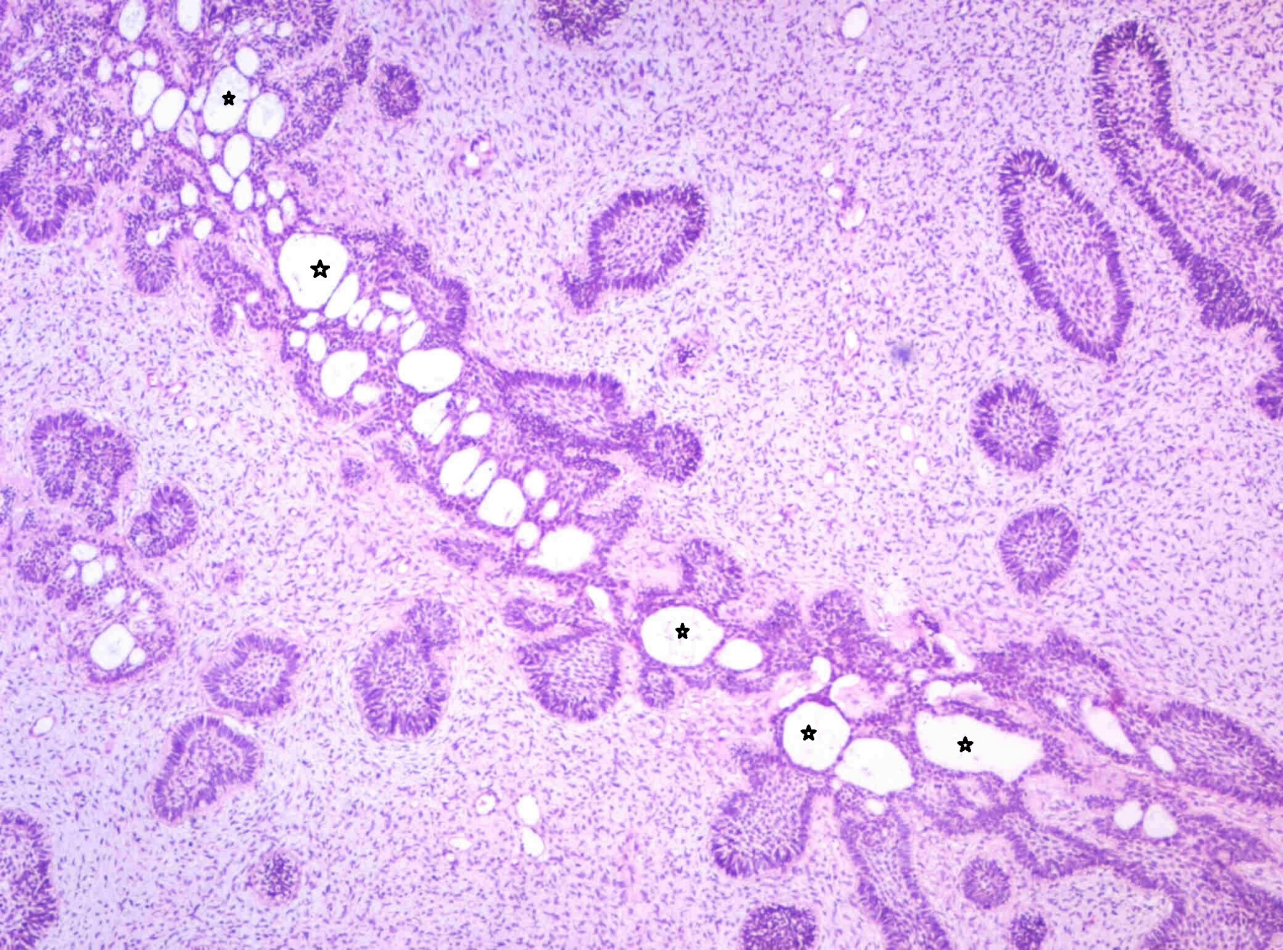
Photomicrograph showing follicles of odontogenic epithelium with few of them showing central cystic degeneration (Star) (Haematoxylin & Eosin stain, x40).

**Figure 8:**
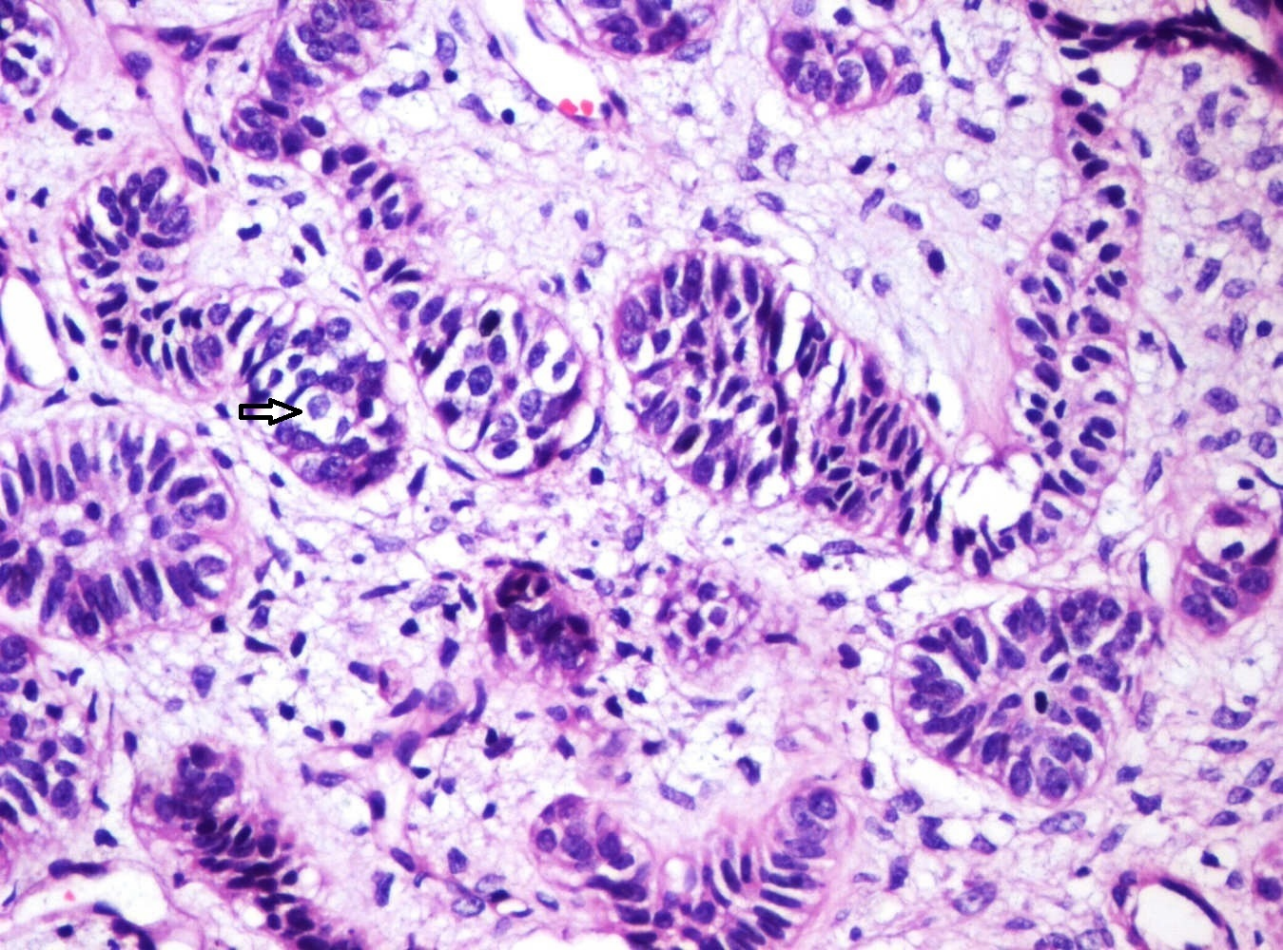
Photomicrograph showing follicles of odontogenic epithelium with few clear cells (Arrow). (Haematoxylin & Eosin stain, x200).

**Figure 9:**
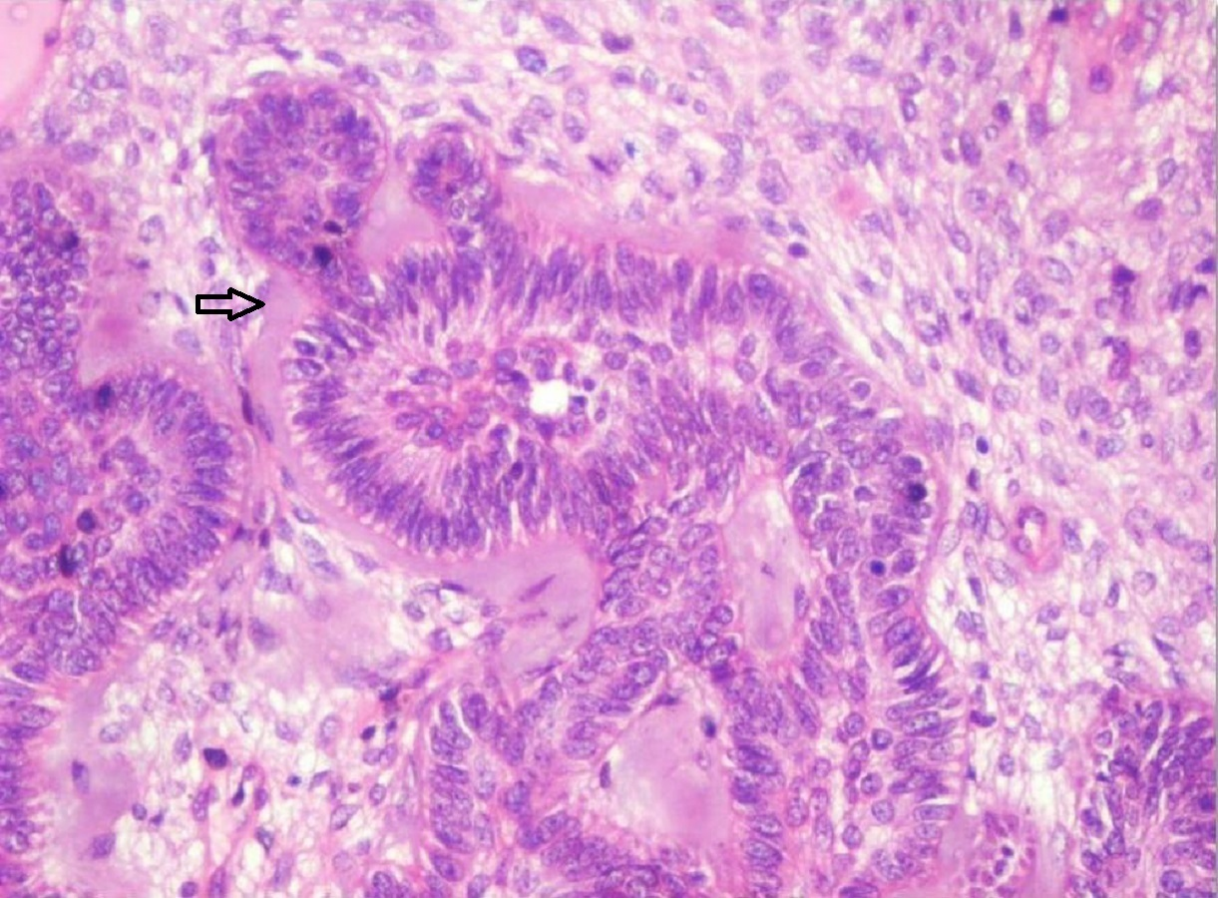
Photomicrograph showing follicles of odontogenic epithelium surrounded by pale eosinophilic hyalinized area resembling dysplastic dentin (Arrow) in cell-rich ectomesenchyme (Haematoxylin & Eosin stain, x100).

**Figure 10:**
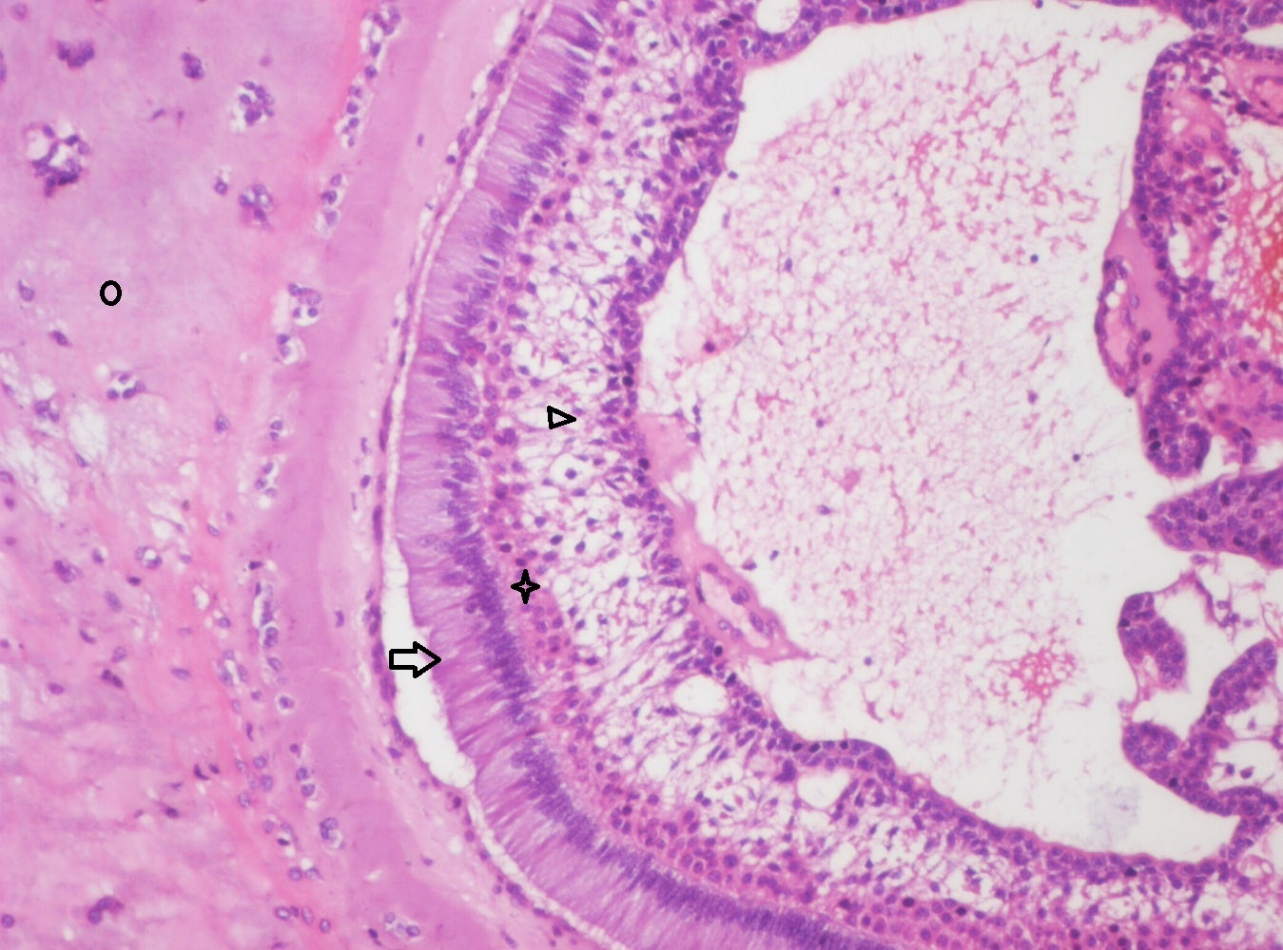
Photomicrograph showing follicle of odontogenic epithelium resembling early bell stage of tooth development consisting of tall columnar cells (Arrow), adjacent flattened layer of cells resembling stratum intermedium (Star) and stellate reticulum like cells (Arrow head). Central cystic degeneration and the enamel organ surrounded by pale eosinophilic hyalinized (Circle) area with entrapped epithelial cells resembling osteodentin is evident (Haematoxylin & Eosin stain, x200).

**Figure 11:**
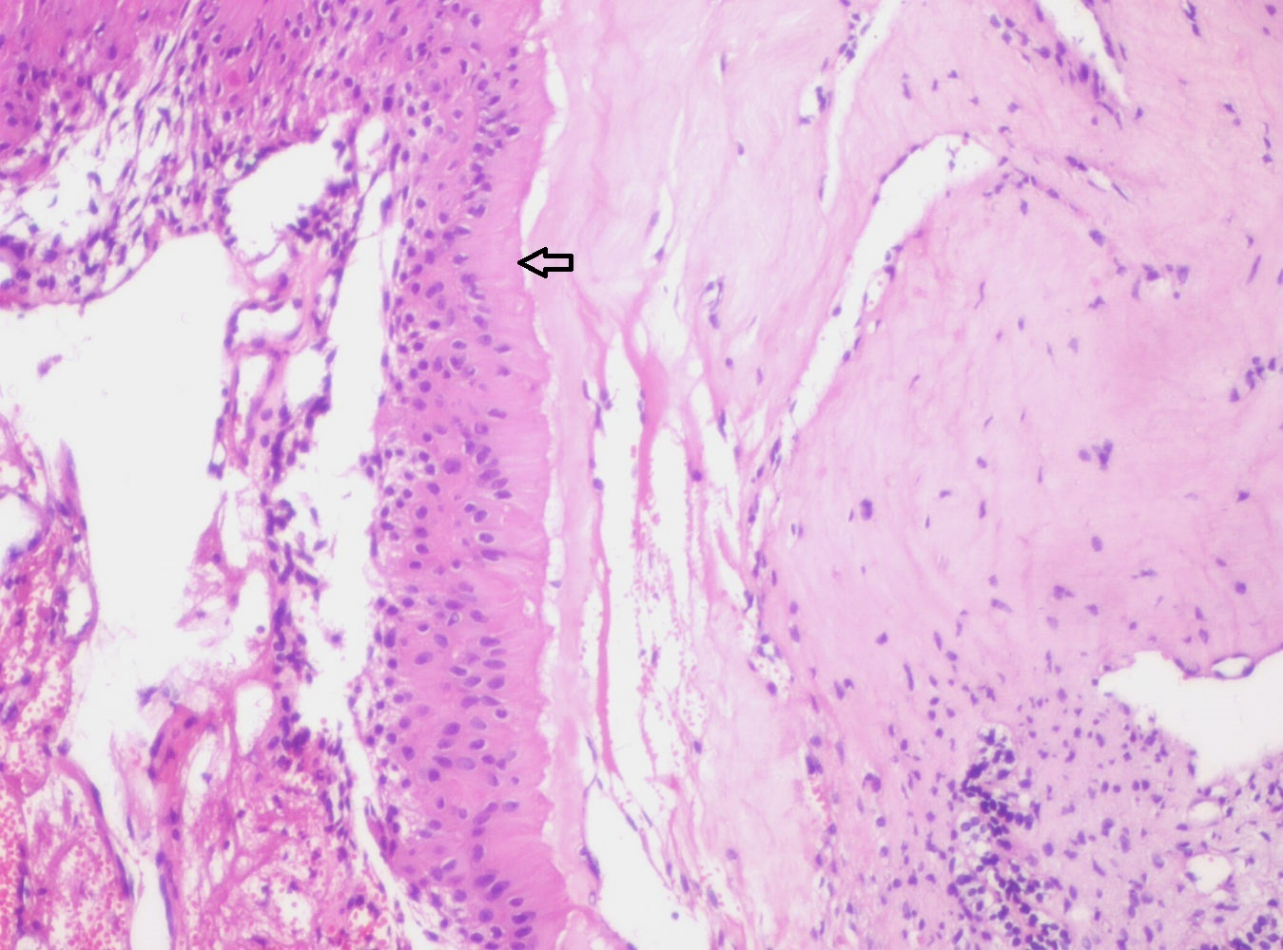
Photomicrograph showing pale eosinophilic hyalinized area adjacent to columnar ameloblast-like (Arrow) cells (Haematoxylin & Eosin stain, x100).

**Figure 12:**
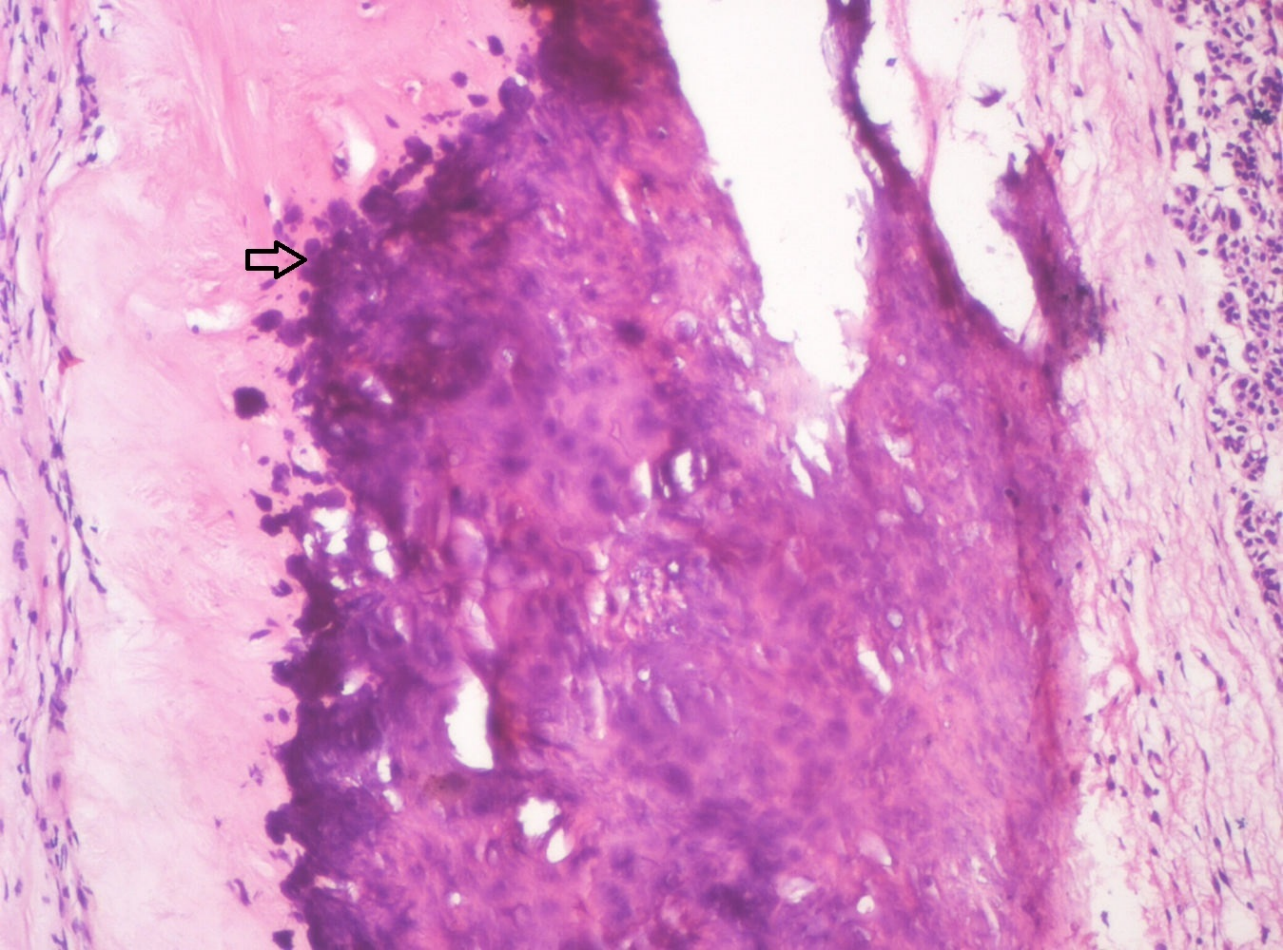
Photomicrograph showing hyalinized eosinophilic area with basophilic globular (Arrow) type of mineralization (Haematoxylin & Eosin stain, x200).

**Figure 13:**
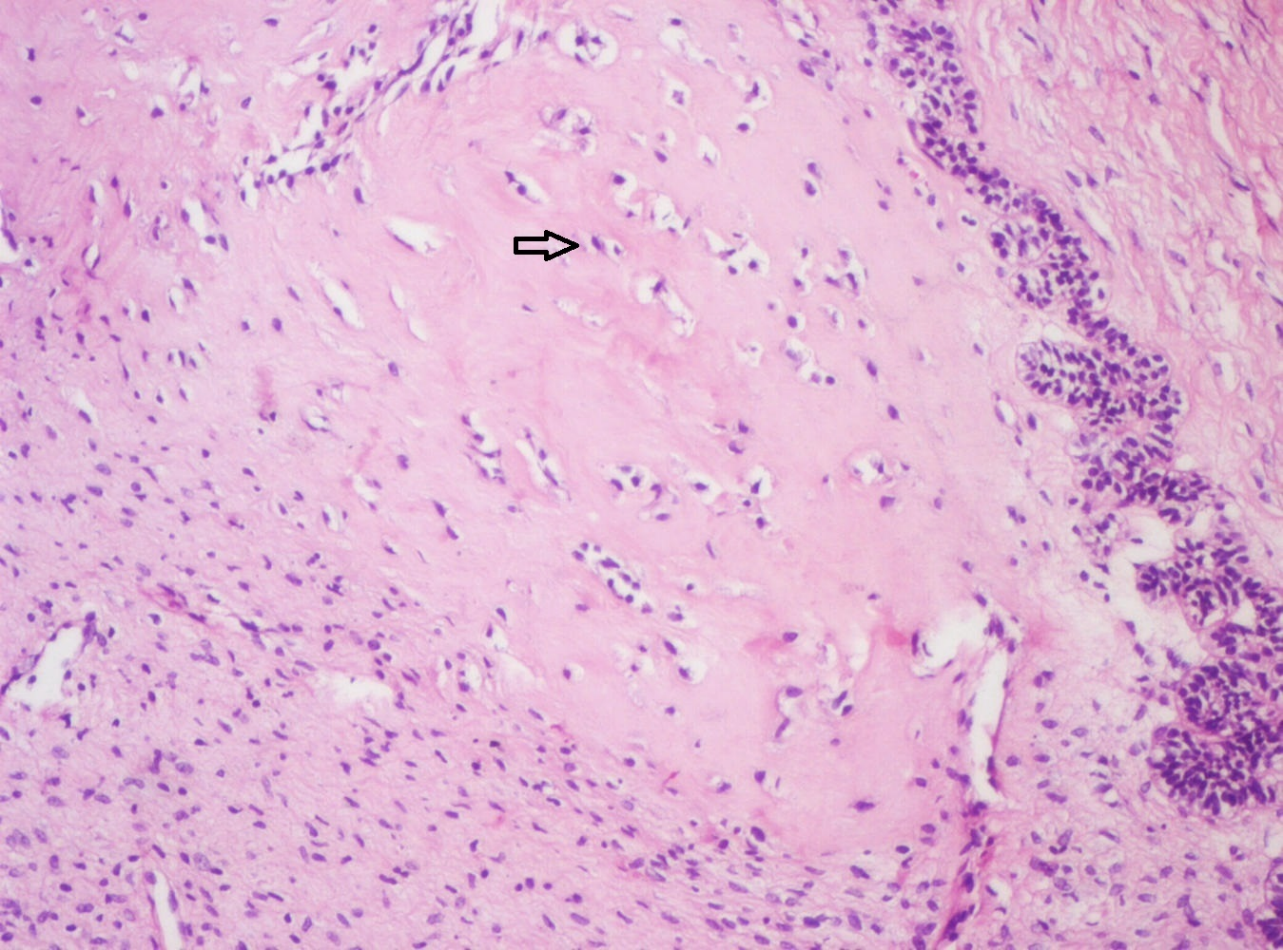
Photomicrograph showing pale eosinophilic hyalinized area with entrapped epithelial cells (Arrow) resembling osteodentin (Haematoxylin & Eosin stain, x100).

Some new findings added enigma to the histopathological findings. Few areas of odontogenic follicles in the ectomesenchyme which had opened showed central cystic degeneration (Figure 7). In the odontogenic epithelial follicles few areas of clear cells were seen (Figure 8). Few follicles were surrounded by halo-like hyalinization (Figure 9). The tumor showed certain areas that were similar to odontogenic apparatus in the bell stage, with large enamel organ like structures. Tall columnar cells similar to ameloblasts, stratum intermedium and stellate reticulum-like cells were seen (Figure 10). Adjacent to the ameloblast-like cells, dysplastic dentin was laid, dentin with predentin-like areas were seen. Induction of dentin was seen in many areas and in many forms. Some eosinophilic areas next to the epithelium with tall columnar cells that had enticed cells similar to osteodentin were seen (Figure 11). The other areas showed large masses of dentin with globular mineralization (Figure 12). Dentinoid-like tissue was also seen with entrapped epithelial cells in few areas adjacent to the odontogenic epithelium (Figure 13).

There was no evidence of enamel or enamel matrix formation even after extensive sectioning, ruling out the diagnosis of AFO. The other differentials considered were ameloblastoma. Though, few odontogenic follicles with peripheral columnar cells and central stellate reticulum like cells were evident, highly cellular ectomesenchyme was also present impressing that the tumor showed proliferation of both epithelial and mesenchymal components, whereas ameloblastoma shows proliferation of only epithelial component and even the conventional reversal of polarity of the ameloblast-like cells was also not seen, thus ruling out ameloblastoma [3].

## Discussion

AFD is a rare neoplasm of odontogenic epithelium with immature ectomesenchyme and formation of dysplastic dentin. For a while it had also been named as a dentinoma [3-4].

Controversy surrounds the categorization of AFDs. The continuum concept with AF, AFO, and odontomas proposed by Cahn and Blum remains disproved on the basis of age of occurrence, site of occurrence, histopathology, gender, and evidence studied in recurrent cases [2-5].

The 2005 WHO classification included AFDs under AFs, but the periostin activity studied by Chau, et al. disapproves this categorization, as AFs did not show increase in periostin activity in the stroma with increasing maturity, and concluded that AFs should be regarded as a true neoplasm whereas AFO is more likely a developing odontoma [6-8]. The recent 2017 WHO classification considers AFDs to be a hamartomatous process based on the consensus that once the hard tissue is formed they mature to form odontomas [1]. However, cases have been reported where AFDs show enhanced growth enlarging to considerable sizes causing cortical plate perforation. There have been reports where recurrence and malignant transformation have been noticed, which show the tumor to have significant growth potential and thus be a true neoplasm [2,4-5]. We propose AFDs to be recognized as an independent tumor entity and acknowledge its rarity.

AFD is very similar to AF and AFO in biological certainty. Generally, a slow growing asymptomatic tumor, in some instances is associated with unerupted teeth and is commonly seen before the age of 20 years with a male predilection, the ratio being 2:1. Most of them are intraosseous tumors in the posterior mandibular region with about 30% showing cortical perforation. It has been observed that AFD in relation to permanent teeth are commonly seen in the posterior region, whereas those in relation to deciduous teeth are seen in the anterior region [2,5]. Radiologically, AFD are well delineated radiolucencies with varying degrees of radiopacity and frequently found in relation to malposed, unerupted teeth [6].

The present case though was seen in a 14-year-old female patient, presented in the mandibular right posterior quadrant involving unerupted 46, 47, and 48 as a unilocular, well-defined radiolucency of approximately 3 x 2 cm, but no such opacity nor cortical perforation was seen. The alveolar ridge superiorly was completely destroyed allowing the tumor to present as a soft tissue mass extending on the alveolar ridge in the region of 43 to 48.

The histopathology of AFD has great resemblance to AF, showing odontogenic epithelium in strands and small islands resembling dental lamina and enamel organ placed in a very cellular primitive ectomesenchyme resembling the dental papillae [4]. Few odontogenic epithelial islands or follicles were lined by tall cuboidal to columnar cells with central stellate reticulum-like cells similar to ameloblastic follicles. Cystic degeneration in the follicles could also be seen [5]. Dentin deposition in relation to the odontogenic epithelium and ectomesenchyme was seen in the form of osteodentin, dentinoid or tubular dentin. Dentin was also seen to entrap the odontogenic epithelium and ectomesenchymal cells. Some areas of hyalinization which may or may not be dentin were also evident [9]. It is important to note that no enamel or enamel matrix was seen in cases of AFD [3].

The present case also showed odontogenic epithelium similar to dental lamina. Small follicles or islands with central stellate reticulum-like cells with few follicles undergoing cystic degeneration were also evident. The ectomesenchyme in which the odontogenic epithelium was arranged was very cellular with plump fibroblasts. Multiple areas showed a halo-like hyalinization around the odontogenic epithelial follicles impressing upon induction, though not bearing resemblance to odontogenic hard tissue. Few areas showed enamel organ in early bell stage like differentiation with no evidence of enamel formation. Different forms of dentin such as osteodentin, dentinoid, and dentin undergoing globular mineralization were noted to be formed in the ectomesenchyme and also in relation to odontogenic epithelium at few areas. Osteodentin-like areas showed matrix with ectomesenchymal cells entrapped in the matrix. At certain places, osteodentin-like areas were bordered by odontogenic epithelium. Large areas with globular mineralization along with unmineralized predentin-like area were seen. Many areas showed non-tubular dentin entrapping cells in multiple lacunae in close relation to odontogenic epithelium resembling dentinoid as reported by Gardner and Farquhar [10]. Most of the inductive material in the form of dysplastic dentin with globular mineralization, osteodentin, and dentinoid were seen close to the periphery whereas a halo lining of hyalinization around many odontogenic epithelial follicles was also a common finding in many areas, as reported by Girish and Garg [9]. No enamel-like material was found in the present case, so ameloblastic fibro-odontome was not considered.

It may be suggested that that all the tissue bearing resemblance to dentin should be considered as odontogenic/dentin induction in normal or dysplastic form. However, hyalinization needs to be categorized as non-dentinal or non-specific induction in the ecto-mesenchyme, the significance of which is not very clear or specific and might be an abortive effect too. More work needs to be done in detailing and recognition of inductive changes in odontogenic tissues and tumors.

## Conclusions

The present case clearly shows a clinical presentation that depicts a destructive tumor. The histopathology showcases a varied amount and variety of dysplastic dentin, validating true odontogenic induction in the tumor. Hence, we think that this case might add value to understand it as a true independent entity among odontogenic tumors.

## References

[1] Wright JM, Vered M (2017). Update from the 4th edition of the world health organization classification of head and neck tumours: odontogenic and maxillofacial bone tumors. Head Neck Pathol.

[2] Chrcanovic BR, Gomez RS (2017). Ameloblastic fibrodentinoma and ameloblastic fibro-odontoma: an updated systematic review of cases reported in the literature. J Oral Maxillofac Surg.

[3] Reichart PA, Philipsen HP (2004). Odontogenic Tumors and Allied Lesions. http://www.quintpub.com/display_detail.php3?psku=B8823#.WR-xJpKGPIU.

[4] Shwetha V, Vineeth K, Prasad K (2016). Ameloblastic fibrodentinoma: a rare occurrence. Int J Med Dent Case Rep.

[5] Jacob O A, Padmakumar S K, Aloka D (2015). Ameloblastic fibrodentinoma: a 12 years follow-up of a rare entity. IJSS Case Reports Rev.

[6] Slootweg PJ (2005). Chapter 6: Odontogenic tumors. World Health Organization Classification of Tumours: Pathology and Genetics of Head and Neck Tumours. 2nd ed.

[7] de Sousa Lopes MLD, Mara Luana Batista Severo MLB, de Souto Medeiros MR (2017). Ameloblastic fibro-odontoma: case report and immunohistochemical profile. J Oral Maxillofac Surg Med Pathol.

[8] Wright JM, Odell EW, Speight PM (2014). Odontogenic tumors, WHO 2005: where do we go from here?. Head Neck Pathol.

[9] Giraddi GB, Garg V (2012). Aggressive atypical ameloblastic fibrodentinoma: report of a case. Comtemp Clin Dent.

[10] Gardner DG, Farquhar DA (1979). A classification of dysplastic forms of dentin. J Oral Pathol Med.

